# The attitudes of neonatologists towards extremely preterm infants: a Q methodological study

**DOI:** 10.1136/archdischild-2014-308071

**Published:** 2015-07-15

**Authors:** Katie Gallagher, Narendra Aladangady, Neil Marlow

**Affiliations:** 1 Florence Nightingale School of Nursing and Midwifery, King's College London, London, UK; 2 Neonatal Unit, Homerton University Hospital NHS Foundation Trust, London, UK; 3 Centre for Paediatrics, Barts and the London School of Medicine and Dentistry, Queen Mary University of London, London, UK; 4 UCL Elizabeth Garrett Anderson Institute for Women's Health, University College London, London, UK

**Keywords:** Neonatology, Intensive Care, Ethics

## Abstract

**Objectives:**

The attitudes and biases of doctors may affect decision making within Neonatal Intensive Care. We studied the attitudes of neonatologists in order to understand how they prioritise different factors contributing to decision making for extremely preterm babies.

**Design:**

Twenty-five neonatologists (11 consultants and 14 senior trainees) participated in a Q methodological study about decision making that involved the ranking of 53 statements from agree to disagree in a unimodal shaped grid. Results were explored by person factor analysis using principle component analysis.

**Results:**

The model of best fit comprised 23 participants contributing a three-factor model, which represented three different attitudes towards decision making and accounted for 59% of the variance. Fourteen statements were ranked in statistically significant similar positions by 23 participants; consensus statements included placing the baby and family at the centre of care, limitation of intervention based upon perceived risk and non-mandatory intervention at birth. Factor 1 participants (n=12) believed that treatment should not be limited based on gestational age and technology should be used to improve treatment. Five factor 2 participants identified strongly with a limit of 24 weeks for treatment, one of whom being polar opposite, believing in treatment at all costs at all gestations. The remaining six factor 3 participants identified strongly with statements that treatment should be withheld on quality of life grounds.

**Conclusions:**

This study has identified differences in attitudes towards decision making between individual neonatologists and trainees that may impact how decisions are communicated to families.

**What is already known on this topic**

- Survival at extremely low gestational ages shows national and regional variation for reasons that are rarely evident
- Nurses show significant variation in attitudes to this patient group that could affect how decisions to institute or continue care may be perceived

**What this study adds**

- Neonatologists agree that treatment may be limited based on perceived risk.
- Neonatologists show significant variation in approach to their interpretation of risk for extremely preterm babies that may colour their attitude to decision making with families.

Despite improvements in neonatal survival and outcome, death following extremely preterm birth remains relatively common: during 2006 in England, mortality rose from 22% at 26 weeks gestation to 78% at 23 weeks.^1^ The majority of deaths in neonatal intensive care are ‘planned’: a decision is made to redirect care from intensive to supportive, or palliative, care and approximately 60%–80% of all deaths in the neonatal unit occur following the withdrawal of life sustaining treatment.^2^ Among extremely preterm infants born in England during 2006, 76% of 580 neonatal unit deaths were described as ‘planned’^2^ and therefore followed conversations about the direction of care.

National recommendations support healthcare professionals in making difficult resuscitation decisions for extremely preterm infants,^3–5^ but a recent survey of practices throughout south east England highlighted that neonatologists frequently reported attitudes in direct contrast with recommendations.^6^ We recently surveyed practices across Europe: reported policy ranged from the neonatologist having full independent decision-making capacity, through parent-led decision making, to guidelines which dictate full resuscitation regardless of infant condition at birth.^7^ Falling mortality is accompanied by an increased likelihood of resuscitation together with significant reduction in the upper limit of discretionary non-resuscitation,^8^ although practice varies widely between individual hospitals, for example, in the National Institute of Child Health & Human Development Neonatal Network.^9^ Thus, in high-income countries there is little consistency or agreement regarding initiation of treatment for extremely preterm infants, with resulting implications for a range of outcomes.

Local practices and national policy are an important factor in determining neonatologist preferences for resuscitation at extreme preterm gestations,^7^ ^10^ ^11^ but personal beliefs may provide an important bias in practical decision making.^10^ Neonatal nurses are also critical to this process, providing information and emotional support to parents inside and outside of formal discussions.^12^ Attitudes of nurses and doctors may differ with respect to ethically sensitive areas such as continuation of life-sustaining treatment and interpretation of adverse outcomes.^13–15^ In a recent study using Q methodology, we highlighted differences in the attitudes of neonatal nurses to such issues and to parental involvement in decision making at extremely preterm gestations.^15^ Such variability within and between healthcare groups may indicate the potential for conflict between professional groups and for parents who may perceive differences in emphasis from individual members of the healthcare team.

While various factors may affect the resuscitation practices of neonatologists, research has not been undertaken to explore how they prioritise these factors in decision making for preterm infants. The aim of this study was to explore the attitudes and perceptions of doctors working in neonatology towards extremely preterm infants, by exploring how they prioritise different factors associated with decision making, such as parental involvement and adverse outcomes.

## Methods

Q methodology explores participant subjectivity through a process known as Q sorting.^16^ This involves participants ranking a set of statements, derived from the relevant published literature, from agree (+6) to disagree (−6) on a response grid.^17^ The preferences of each participant are entered into a computer software package, PQMethod,^18^ to undertake factor analysis with either principle component or centroid analysis. Factor analysis in Q methodology differs from traditional factor analysis, however, as the rows, rather than the columns, constitute the variables. This results in the participants themselves, rather than the statements, being analysed: ‘by person factor analysis’.^19^ In this way the analysis allows for clusters of attitudes towards the phenomenon under debate to be revealed.^20^ Following rotation, each factor is merged to form a factor array. This uses the original values in the Q sorting process (+6 to −6) to highlight the positioning of statements in a ‘model Q sort’, which represents how a hypothetical respondent with a 100% loading on that factor would have ranked the statements.^21^ Each model Q sort is likely to comprise ‘consensus statements’, which all participants have placed in a statistically significant similar position, and ‘distinguishing statements’, those that participants have placed in a significantly different position to other model Q sorts. The number of distinguishing factors reflects the number of factors retained for rotation. Ranking statements in this way allows for the exploration of attitudes of participants towards a phenomenon, providing information about the perspective itself based on the positioning of statements.

The statements for this study had been previously used to explore the attitudes of neonatal nurses towards extremely preterm infants.^15^ Fifty-three statements were developed from thematic analysis categorised into six themes: (1) adverse outcomes, (2) controversy with abortion limitations (within the UK abortion is permitted until 24 weeks of gestation^22^), (3) decision-making involvement, (4) technology used to promote survival, (5) treatment decisions themselves and (6) issues around fertility in relation to neonatology.

Experienced neonatal doctors (Consultants and Specialty Registrar ≥4 years) from two tertiary level neonatal units in London were invited to participate in the Q study between May and November 2012. In total, 25 volunteered participation. Following written consent, participants were invited to rank order the statements from agree (+6) to disagree (−6) in a unimodal distribution response grid. Q sort completion took 40–60 min. The Research and Development Committees of the two participating hospitals approved the study. On advice, research ethics committee permission was not deemed necessary.

### Data analysis

Q sorts were entered into PQMethod^18^ for analysis. Factor analysis of the participants was undertaken using principle component analysis, which produced a number of factors with eigenvalues over 1. Each factor solution was subjected to varimax rotation to determine the best solution for the data, that is, how many factors to retain for analysis.^23^ We chose the solution in which the number of factors was balanced by inclusion of the majority of the participants; retaining too few or too many factors resulted in the exclusion of participants from the results with the additional loss of distinguishing statements, preventing interpretation of the model Q sorts.

## Results

Five and four-factor solutions explained 69% and 64% of the variance but only 16 and 20 participants, respectively, had significant loadings on the resulting factors (p<0.01). A three-factor solution resulted in the inclusion of 23 of the 25 participants (p<0.01), accounted for 59% of the variance and had a suitable number of distinguishing statements to allow for analysis; thus, this model was chosen to represent the data. From the data set, we derived a set of consensus statements and three distinguishing factors; 12 individuals reflected factor 1, five factor 2 and six factor 3.

### Consensus statements

Of the 53 statements, 14 (26%) were placed in a statistically significant similar position by the 23 participants (table 1).

**Table 1:** Q sort factor array: consensus statements (mean statement positions for individuals contributing to factors (F) 1, 2 and 3)

Consensus statements	F1	F2	F3
Evidence of severe disability is a valid reason to withdraw treatment in an extremely preterm infant*	+5	+4	+6
The technology which enables the most premature of infants to survive brings with it increased ethical dilemmas over whether it should be used to ensure this survival*	+4	+6	+3
The care of women in the neonatal unit should not be influenced by a history of previous abortions	+5	+4	+3
If life-limiting disability is diagnosed prenatally, parents should be able to give birth to their child and enjoy the time they have without the option of full intensive care treatment*	+4	+5	+2
The technology used on the neonatal unit allows more safety and control as the infants status is continually updated	+4	+2	+5
The most important factor when deciding on resuscitation is the potential of long-term suffering to the baby	+3	+3	+4
Health care professionals (HCP) who work in abortion services from 20 to 24 weeks of gestation are merely providing a service and should not be judged*	+2	+3	+2
Full intensive care treatment should always be started as it can be withdrawn later if found to be futile	−1	−2	−1
Infant survival has become a secondary outcome, with determining how far technology can advance survival limits seemingly more important	−2	−1	−1
Caring has become technological, shifting the focus from caring from the infant to caring for the technology	−2	−1	−1
Infants born extremely preterm to families who have received in vitro fertilisation and unlikely to conceive again should always be offered full intensive care treatment at all costs*	−4	−3	−2
HCP should deliver the care that parents ask for, even if parents are asking for treatment that HCP think is futile	−4	−4	−4
Parents should not be involved in treatment decisions for extremely preterm infants as they do not understand complex medical information*	−5	−3	−4
Life should be maintained irrespective of outcome	−5	−6	−5

All factors p<0.05.

*Factors with p<0.01.

The positioning of statements reflected the opinion that while decision making for preterm infants should be infant-centred and involve parents, there should be a limitation to treatment based upon perceived adverse outcomes for the infant. Further statements reflected the participants’ perception that while technology is important in ensuring the safety of treatment, it should not automatically be used as it can raise ethically challenging situations.

### Factor 1: distinguishing statements

The Q sorts of 12 participants loaded onto a factor defined by 18 statements accounting for 27% of the total variance and with high reliability (0.98; table 2).

**Table 2:** Mean level of consensus for statements by factor 1 participants (mean levels of consensus for statements by factor 2 and 3 participants in parentheses for comparison)^13^

Factor 1 (n=12)	F1	(F2, F3)
Death is, and always will be, inevitable for some infants	+6	(3, 3)
Peaceful death is more important than full intensive care treatment	+5	(2, 1)
Better provision of community services once children are older would make it easier to continue treatment for extremely preterm infants who display evidence of disability	+3*	(1, 0)
Technology should be advanced to allow the most premature of infants to survive	+2	(−1, 4)
There is a cross-over between neonatal and abortion services as both care for women at similar gestations	+1	(4, 4)
Advancing technology has made the process of withdrawing treatment more difficult	+2	(4, 4)
Technological developments mean that heroic measures of extraordinary means of support are overused	+1*	(2, 5)
Abortion providers and neonatal units are separate entities and the actions of one should have no influence upon the other	0*	(2, 2)
The amount of technology used in the neonatal unit is a barrier which is detrimental to parent–infant bonding	0*	(1, −2)
Infants born extremely preterm with life-limiting illness should still be given full intensive care treatment	0*	(−3, −5)
Women should have the right to choose abortion up until 24 weeks of gestation	0*	(5, −2)
Attempting to save infants <24/40 weeks is a large uncontrolled experiment	−1	(−6, −5)
‘Infants’ who are born alive following termination of pregnancy should be transferred to the neonatal unit for a trial of life	−1	(−6, −5)
Saving infants <24/40 weeks is an inefficient use of NHS resources	−2	(2, 3)
Neonatal unit treatment accounts for a large proportion of NHS resources and as such admission of infants <24/40 weeks should be restricted	−3	(0, −1)
Older parents are better equipped to deal with the outcome of extreme prematurity	−3*	(0, −1)
It is wrong to knowingly bring a disabled child into this world	−5	(−1, 2)
Life satisfaction is not possible if you have a disability	−6	(−3, −1)

All factors <0.05.

*Factors with p<0.01.

NHS, National Health Service.

Statements dominating this factor represent treatment issues and technology (six and five statements, respectively). Participants prioritised statements that indicate an acceptance of death in certain scenarios; however, they were clear that treatment should not be limited based on gestational age. Statements also highlight a positive reaction to the idea that technology should be advanced to help improve treatment, if areas such as community support for infants and families with complex needs are addressed.

### Factor 2: distinguishing statements

The Q sorts of five participants loaded onto a second factor defined by 16 statements, accounting for 15% of the total variance, with high reliability (0.95; table 3).

**Table 3:** Mean level of consensus for statements by factor 2 participants (mean levels for statements by factor 1 and 3 participants in parentheses for comparison)^13^

Factor 2 (n=5)	F2	(F1, F3)
The current abortion limit of 24 weeks of gestation is adequate, as infants <24/40 weeks should not normally be resuscitated due to low survival rates and high risks of disability	5	(−1, 0)
Women should have the right to choose abortion up until 24 weeks of gestation	5	(0, −2)
The amount of technology surrounding the infant alters the concept of death to something that can be overcome	3	(1, 1)
Technological developments means that heroic measures of extraordinary support are overused	2*	(1, 5)
The amount of technology used in the neonatal unit is a barrier which is detrimental to parent–infant bonding	1*	(0, −2)
Parents should be shown morbidity and mortality statistics following preterm birth to help facilitate decision making	0	(3, 2)
The more disabilities that can be diagnosed prenatally, the more pressure there is on women to abort these pregnancies	0	(1, 1)
The choices that parents make about their extremely preterm infant are often prompted by the choices of the health care professionals	0*	(2, 1)
Euthanasia protocols for extremely preterm infants should be introduced in the UK	−1	(−4, −3)
It is wrong to knowingly bring a disabled child into this world	−1	(−5, 2)
Technology should be advanced to allow the most premature of infants to survive	−1*	(2, −4)
Always initiating full intensive care treatment gives parents a chance to think that they have done everything they possibly could	−2	(0, 1)
Infants born extremely preterm with life-limiting illness should still be given full intensive care treatment	−3*	(0, −5)
Life satisfaction is not possible if you have a disability	−3	(−6, −1)
The abortion limits should be reduced in acknowledgement and accordance with the current limits of viability	−4	(1, 0)
Abortions should not be allowed from 22/40 weeks as the fetus is changing into a baby	−5	(−1, 0)

All factors <0.05.

*Factors with p<0.01.

Statements dominating this factor represented abortion, disability and technology (four statements, respectively). Of these five individuals, one participant disagreed with the opinions expressed in this factor (ie, was the reverse of the other four participants), indicating a strong belief in treatment at all costs and disagreement with abortion. Among the remaining participants, the positioning of statements reflected the perspective that treatment should be limited to infants at or >24 weeks of gestation, agreement with the abortion limit at 24 weeks of gestation and a belief that resuscitation should not be initiated if there is evidence of disability (although paradoxically participants were clear they believed life satisfaction is possible with a disability). These distinguishing statements may be considered more paternalistic: participants placed the importance of showing parents mortality and morbidity statistics in the neutral position.

### Factor 3: distinguishing statements

The Q sorts of six participants loaded onto a third factor, defined by 16 statements, accounting for 15% of the total variance with high reliability (0.96; table 4).

**Table 4:** Mean level of consensus for statements by factor 3 participants (mean levels of consensus for statements by factor 1 and 2 participants in parentheses for comparison)^13^

Factor 3 (n=6)	F3	(F1, F2)
Technological developments mean that heroic means of extraordinary means of support are overused	5	(1, 2)
Parents who do not want a disabled child should be able to make the decision to withhold or withdraw full intensive care treatment	5	(1, 1)
It is wrong to knowingly bring a disabled child into this world	2	(−5, −1)
Resuscitation at <24/40 weeks is for the parents benefit, not for the infants	1	(−2, −1)
The most important factor when deciding on resuscitation is the parents decision	1	(−1, −1)
Babies born at <24/40 weeks gestation should always be resuscitated if the mother is too old to have any more children	0	(−3, −5)
Parents are given a false hope when they see all of the equipment used on their extremely preterm infant	−1	(2, 1)
Life satisfaction is not possible if you have a disability	−1	(−6, −3)
Women who try to conceive post menopause are not thinking about the best interests of the infant	−1	(2, 1)
The philosophy underpinning nursing and medical care is the same in all healthcare settings, including neonatal and abortion services	−2*	(0, 1)
Women should have the right to choose abortion up until 24/40 weeks	−2*	(0, 5)
The amount of technology used in the neonatal unit is a barrier which is detrimental to parents infant bonding	−2	(0, 1)
Deciding whether to withhold or withdraw treatment is too stressful for parents and should be done by the health care professionals	−3	(0, 0)
Technology should be advanced to allow the most premature of infants to survive	−4*	(2, −1)
Infants born extremely preterm with life-limiting illness should still be given full intensive care treatment	−5*	(0, −3)
It is better to have a disabled child, no matter how disabled, than no child at all	−6*	(−2, −4)

All factors <0.05.

*Factors with p<0.01.

Dominating statements were around disability and technology. These individuals had a strong perception that disability is a valid reason to withhold or withdraw treatment due to quality of life concerns, and that parents should have a voice in this decision. Participants perceived technology as something which can help preterm infants but felt strongly that its application was overused as a heroic means of extraordinary support in the neonatal unit.

## Discussion

Although there was consensus that the perceived risk of disability in an infant should limit the treatment options available, the participants were divided around using risk of impairment or gestational age to limit treatment and the importance of involvement of parents in this decision-making process. In these groupings there were striking parallels with our findings among 36 neonatal nurses from a different region of the UK.^15^

Although our study is limited through its relatively small sample size, it is of similar size to many investigations in qualitative research. A further sample should be recruited to understand whether we have reached saturation within the opinions of this professional group.

While many studies in the literature have shown differences in practices between physicians at extremely preterm gestations,^9^ ^11^ ^14^ ^24^ this study has highlighted some of the potential reasons for these differences. Attitude has long been suggested as a major influence behind variations in these practices but with little evidence. Doctors in this study were able to articulate these ethically complex attitudes allowing us to quantify how attitudes may underpin elements of personal practice.

Throughout Europe, studies show large variations in survival rates for infants born 22–25 weeks of gestation.^2^ ^25^ ^26^ Even within countries, outcomes for infants born at these gestations may vary from region to region, as national guidelines are not binding for practice and allow for personal interpretation.^27^ It is interesting to speculate the extent to which personal beliefs may underpin some of this variation. For some time there have been declared regional variations in practice in Sweden^25^ because of differing senior professional attitudes towards active care. Very high levels of survival at 23 and 24 weeks were reported from the national EXtremely PReterm infants Study in Sweden Study,^28^ but there remains significant regional variation in outcome^25^ and practice.^29^ In contrast to mortality, morbidity rates appear to be similar across regions and overall very similar to levels reported in EPICure: Extremely Preterm Infants Cure2, which has much lower survival.^30^ A similar effect was noted in Denmark where a change in attitude from minimal intervention to proactive management for infants born <26 weeks resulted in a significant increase in the numbers of survivors but no change in the proportion with morbidity.^31^ Khan *et al*^27^ suggests that ‘attitude is a little thing that makes a big difference’ on outcomes for this group of infants, and a variation in approach that appears to be unique, when compared with any other age.

Variations in practice based upon the attitudes of individual physicians have a number of consequences that need to be considered and are not recorded. From a research perspective, it is virtually impossible to compare outcomes from different regions or countries without understanding the differences in approach.^32^

Most delivery room decision making is based primarily upon gestational age, as demonstrated in this study. Other factors will also contribute towards risk, for example, birth weight, plurality, infant sex and the use of antenatal steroids.^33^ However, the fixed parameters of weight and sex provide only a small additional predictive ability, compared with the enormity of the decision to intervene or not, and the use of steroids seems only logical when active support is being planned. Given that proactive management at birth can improve survival without seemingly impacting morbidity, it is critically important to know the degree of active intervention when interpreting survival data, something rarely explicitly reported.^9^ ^34^ ^35^

What often underpins practice is an individual's perception of the impact of adverse outcomes that may follow the provision of treatment below their preferred gestational age.^26^ ^36^ This perception does not always match the information widely available from population studies,^37^ and thus there may be a tendency to emphasise impairments that do not cause ‘severe’ disability. Doctors with such bias may be less likely to intervene at earlier gestations; poor survival then becomes a ‘self-fulfilling prophecy’. It is clear from this and other studies that survival and disability do not hold consistent meaning for parents and healthcare professionals.^38^ ^39^ We have demonstrated varying attitudes among neonatologists to treatment options dependent upon personal perception of outcomes.

The personal opinions of the physicians are likely to have a different reference framework to those of parents. Previous research has shown that compared with neonatal team members, parents are likely to be more optimistic regarding the outcome of disability in their infant and are less likely to see a disabled outcome as a ‘fate worse than the death of their infant’.^40^ Such differences in perceptions can have implications for the interactions between the healthcare team, the parents and individual professionals and for the level of parental involvement in decision making.

Our previous research using the same methodological approach highlighted that neonatal nurses also have wide variation in their attitudes towards extremely preterm infants.^15^ They do, however, share a similar consensus with doctors who perceived that outcomes of disability should be an important factor in limiting treatment for extremely preterm infants. This adds a further layer of complexity to a situation where parents have reported nurses as being crucial to their ability to make informed decisions.^12^ An unshared assumption about the perception of such adverse outcomes may prevent consensual decision making between parties and could promote a feeling of non-involvement on the part of parents decreasing their satisfaction with the decision-making process.

In a recent exploration of parental decision making in Canada, parents expressed their approach through a variety of ways, including decision making as a series of consequences for both parents and the infant, and indecision as a decision in itself.^41^ In a further study parents expressed their beliefs that guidelines make decision making easier for physicians, but not for parents.^42^ Parents were strongly of the opinion that they should be involved in writing practice guidance so that the parental perspective was not lost. Evidence suggests that this is rare in Europe.^7^ Even within the UK, which has included parent groups in guidance development,^4^ ^43^ parents often perceive less than optimal involvement in decision making and communication with doctors about their child; many parents report being distressed by insensitive communication or overwhelmed by conflicting information.^44^ Research into the dynamics of this critical decision-making process is required, to uncover how attitudinal preferences are expressed (and considered) among neonatal staff and parents, as expression of attitude alone only implies, and does not determine, action.

This study has provided an indication of the underlying attitudes of healthcare professionals towards decision making for extremely preterm infants. Further research is required to explore the relationship between neonatologists’ attitude and practice, through determination of the resulting infant outcomes, and including practices in both delivery room and neonatal unit. Analysis of interactions between parents and the neonatal team during conversations about decision making would add further insight into how these attitudes are managed in practice.
